# Respiratory Syncytial Virus and COVID‐19 in Hospitalized Adults in Spain: Clinical, Radiological Features and Antimicrobial Use

**DOI:** 10.1002/iid3.70281

**Published:** 2025-11-25

**Authors:** Jose‐Reynaldo Homen Fernandez, Inés Armenteros, Adrián Valls Carbó, Julia Barrado, Carolina Olmos‐Mata, Ana Muñoz, Juncal Pérez‐Somarriba, Noemí Cabello, María José Núñez, Vicente Estrada

**Affiliations:** ^1^ Servicio de Enfermedades Infecciosas, Instituto de Investigación Sanitaria del Hospital Clínico San Carlos (IdISSC) Hospital Clínico San Carlos Madrid Spain; ^2^ Centro de Investigación Biomédica en Red de Enfermedades Infecciosas (CIBERINFEC), Instituto de Salud Carlos III Madrid Spain; ^3^ Centro Sanitario Sandoval Hospital Clínico San Carlos Madrid Spain

**Keywords:** antimicrobial stewardship, comorbidity, COVID‐19, epidemiology, hospitalization, inflammatory markers, radiography, respiratory syncytial virus infections, SARS‐CoV2, Spain

## Abstract

**Background:**

Lower respiratory tract infections (LRTIs) impose a significant global burden, with over 400 million cases annually. This study compares the clinical features of adults hospitalized with respiratory syncytial virus (RSV) and COVID‐19, two viral pathogens with similar presentations but differing epidemiology.

**Methods:**

This cross‐sectional study analyzed 100 adult cases with PCR‐confirmed RSV or COVID‐19, admitted to the hospital from January 2022 to March 2023. Data on clinical, sociodemographic, radiological, treatment, and laboratory variables were extracted from records.

**Results:**

Both cohorts consisted of elderly patients (> 70 years) with multiple comorbidities. Notably, the RSV group had a higher prevalence of CHF (24% vs. 10%, *p* = 0.014) and COPD (29% vs. 9%, *p* = 0.001). Radiologically, 51% of RSV patients had normal findings, whereas 48% of COVID‐19 patients exhibited bilateral pneumonia (*p* = 0.001). Antimicrobial treatment was administered to 75% of RSV patients compared to 41% of COVID‐19 patients (*p* < 0.001). RSV patients had marginally higher leukocyte and neutrophil counts, while COVID‐19 patients showed significantly elevated CRP, ferritin, LDH, ALT, and potassium levels.

**Conclusions:**

Distinct profiles were identified between hospitalized RSV and COVID‐19 patients. RSV patients, mostly older with CHF and COPD, were more likely to receive antibiotics, possibly reflecting the lack of targeted therapies. In contrast, COVID‐19 patients exhibited higher inflammation and lung involvement. These findings highlight the need to refine treatment protocols, enhance antimicrobial stewardship, and develop specific RSV therapies alongside preventive strategies for high‐risk groups.

## 1 Introduction

Lower respiratory tract infections (LRTIs) pose a significant challenge to healthcare systems, with more than 400 million cases reported worldwide in 2019 [1, 2]. Among the described aetiologies, two pathogens have recently gained prominence: respiratory syncytial virus (RSV), identified as the third leading cause of LRTIs in adults [3], and SARS‐CoV‐2, the virus responsible for the COVID‐19 pandemic.

RSV frequently affects children, although infections can occur at any age. In adults with LRTIs, the most common symptoms include productive cough, respiratory distress, general malaise, and fever. Elderly individuals, immunocompromised patients, and those with chronic conditions such as chronic obstructive pulmonary disease (COPD), congestive heart failure (CHF), and diabetes mellitus (DM), among others, are more likely to experience a more severe disease course, with an increased risk of hospitalization and mortality [4, 5].

RSV typically follows a seasonal outbreak pattern, occurring in the Northern Hemisphere from October or November to April or May, with a peak in January or February [1, 3]. However, the COVID‐19 pandemic and associated control measures, such as social distancing and mask usage, led to a decline in RSV circulation and other respiratory viruses, thereby altering their epidemiological patterns [6]. In the years following the peak of the pandemic (post‐2022), both viruses have been circulating simultaneously.

It is important to highlight that RSV and SARS‐CoV‐2 infections present with similar symptoms, making them clinically indistinguishable. Both infections have worse outcomes in older adults and individuals with comorbidities. In this context, the advancements and widespread implementation of molecular diagnostics, which have gained prominence following the pandemic, enable the identification of the causative agent and facilitate the etiological diagnosis of LRTIs [7].

The objective of this study is to compare the clinical characteristics—specifically the prevalence of comorbidities—of adults hospitalized with RSV infection to those hospitalized with COVID‐19, as well as differences in clinical presentation, disease severity, radiological findings, and laboratory results.

This study will contribute to a better understanding of respiratory infections in adults and will provide valuable insights into the diagnosis and clinical management of hospitalized patients with these conditions. Furthermore, it will offer relevant information for identifying vulnerable populations that may benefit from preventive strategies against RSV infection in adults.

## 2 Patients and Methods

### 2.1 Study Design and Study Population

This is a cross‐sectional observational study of a series of adult patients hospitalized at Hospital Clínico San Carlos in Madrid with a confirmed diagnosis of RSV or COVID‐19 infection by polymerase chain reaction (PCR) from January 2022 to March 2023. The first 100 cases of each infection were consecutively selected.

The investigation was reviewed and received scientific approval from the Research Ethics Committee of Hospital Clínico San Carlos (Code 23/450‐E). The Committee granted a waiver of informed consent, considering the study's historical cohort design and the minimal risk posed to participants. As some patients may have passed away or could not be contacted due to the time elapsed since hospitalization, obtaining informed consent was deemed impracticable. Requiring consent in these circumstances could have significantly hindered the feasibility of the study and introduced selection bias, potentially compromising the validity and generalizability of the findings. The study was conducted in accordance with the principles of the Declaration of Helsinki and institutional data protection regulations.

The sample size was calculated based on previously published data comparing comorbidity prevalence in hospitalized patients with SARS‐CoV‐2 and RSV infection. According to a study by Hedberg and colleagues (PMC8260304), the prevalence of any comorbidity was 59% in the SARS‐CoV‐2 group and 82% in the RSV group. Assuming a two‐tailed *α* level of 0.05 and a power of 80%, a minimum of 60 patients/group was required to detect a statistically significant difference. To increase statistical power and ensure robustness of the analysis, we decided to include 100 patients/group, resulting in a total sample size of 200 patients.

### 2.2 Variables

Clinical and sociodemographic characteristics were obtained from the patients' medical records, including age, sex, comorbidities, vaccination status, symptom onset date, length of hospitalization, discharge or death, chest X‐ray pattern (as indicated by the treating physician) and treatment received. Additionally, the following laboratory parameters from the first blood test were documented: hemoglobin, platelets, total leukocytes, absolute neutrophils, absolute lymphocytes, absolute monocytes, thrombin time, prothrombin time, D‐dimer, fibrinogen, blood glucose, total bilirubin, creatinine, estimated glomerular filtration rate (eGFR), urea, alanine aminotransferase (ALT), aspartate aminotransferase (AST), alkaline phosphatase, gamma‐glutamyl transpeptidase (GGT), lactate dehydrogenase (LDH), creatine phosphokinase (CK), sodium, potassium, troponin, C‐reactive protein (CRP), procalcitonin, and ferritin.

### 2.3 Statistical Analysis

Continuous and ordinal variables were described using the mean and standard deviation or the median and interquartile range, as appropriate. Categorical variables were presented as counts and percentages. For group comparisons, the *χ* ^2^ test or Fisher's exact test, when necessary, was applied to categorical variables. Normality of continuous variables was assessed using the Kolmogorov–Smirnov test, followed by comparisons between groups using a *t*‐test, Mann–Whitney test, ANOVA, or Kruskal–Wallis's test, as appropriate. All statistical tests were two‐tailed, and a *p* < 0.05 was considered statistically significant.

To adjust for potential confounding variables, a linear regression analysis was performed, adjusting for age, sex, heart failure, COPD, and antimicrobial use. Statistical analyses were conducted using R version 4.3.1 and RStudio.

## 3 Results

### 3.1 Baseline Characteristics

The characteristics of the patients are shown in Table 1, with both groups consisting of similar populations of older adults over 70 years with multiple comorbidities. A notable difference was observed, patients with RSV had a higher prevalence of CHF (24% vs. 10%, *p* = 0.014) and COPD (29% vs. 9%, *p* = 0.001).

**Table 1:** Demographics and baseline characteristics.

Characteristic	RSV *n* = 100	COVID‐19 *n* = 100	*p*
Age—median [IQR]—yr	83.0 [73.8; 88]	83.5 [75.8; 89]	0.888
Sex—No. (%)			0.157
Female	55 (55)	44 (44)	
Male	45 (45)	56 (56)	
Comorbidities―No. (%)			
Myocardial infarction	9 (9)	10 (10)	1
Congestive heart failure	24 (24)	10 (10)	**0.014**
Dementia	6 (6)	11 (11)	0.310
COPD	29 (29)	9 (9)	**0.001**
Liver disease	4 (4)	1 (1)	0.369
Diabetes mellitus	29 (29)	28 (28)	1
Moderate to severe CKD	19 (19)	14 (14)	0.446
Solid tumor	11 (11)	13 (13)	0.828

*Note:* Bold values indicate statistically significant *p* values (*p* < 0.05).

Abbreviations: CKD, chronic kidney disease; COPD, chronic obstructive pulmonary disease; IQR, interquartile range; yr, year.

### 3.2 Clinical Presentation

The respiratory infection characteristics are summarized in Table 2. The mean length of hospitalization was 7 days in both groups. Low‐flow oxygen therapy was administered to 68% of patients with COVID‐19 and 75% of those with RSV. Regarding imaging studies, 51% of the RSV group had normal findings, whereas 48% of the COVID‐19 group presented a radiographic pattern of bilateral pneumonia (*p* = 0.001). Antimicrobial treatment was administered to 75% of RSV patients compared to 41% of COVID‐19 patients (*p* < 0.001), with beta‐lactams being the most commonly used class. More than 80% of patients in both groups had received at least two doses of the COVID‐19 vaccine. The mortality rate was 14% in the COVID‐19 group compared to 6% in the RSV group (*p* = 0.09).

**Table 2:** Clinical characteristics.

Characteristic	RSV *n* = 100	COVID‐19 *n* = 100	*p*
Hospitalization duration―median [IQR]—days	7 [4; 12]	7 [5; 10]	0.960
Respiratory insufficiency―No. (%)	92 (92.0)	85 (85)	0.184
Mild	75 (85.2)	68 (80)	
Moderate/severe	13 (14.8)	17 (20)	
Death—No. (%)	6 (6)	14 (14)	0.099
Chest radiography pattern—No. (%)			**< 0.001**
Bilateral pneumonia	15 (15)	48 (48)	
Localized pneumonia	17 (17)	12 (12)	
Other alteration	17 (17)	19 (19)	
Normal	51 (51)	21 (21)	
Two or more COVID‐19 vaccinations	91 (91)	83 (83)	0.141
Use of antimicrobial agents	75 (75)	41 (41)	< **0.001**

*Note:* Bold values indicate statistically significant *p* values (*p* < 0.05).

Abbreviation: IQR, interquartile range.

### 3.3 Hematological and Biochemical Parameters

Regarding laboratory parameters, the RSV group exhibited slightly higher levels of total leukocytes (8.7 vs. 7.1, *p* = 0.009) and neutrophils (6.63 vs. 5.18, *p* = 0.002). Conversely, the COVID‐19 group had significantly higher levels of inflammatory and cellular damage markers, with CRP levels of 45.2 versus 20.9 (*p* = 0.004), ferritin levels of 284 versus 133 (*p* = 0.005), and LDH levels of 542 versus 432 (*p* < 0.001). Additionally, the COVID‐19 group showed higher ALT and potassium levels, whereas the RSV group had slightly higher AST and total bilirubin levels. No significant differences were found in the remaining evaluated parameters (Table 3).

**Table 3:** Result of the univariate analysis.

Characteristic	RSV	COVID‐19	*p*
Blood count―median [IQR]			
Absolute leukocytes―×10^3^/μL	8.70 [6.40; 11.1]	7.10 [5.03; 9.17]	**0.009**
Absolute neutrophils―×10^3^/μL	6.63 [4.75; 8.78]	5.18 [3.44; 7.25]	**0.002**
Absolute lymphocytes―×10^3^/μL	0.88 [0.53; 1.24]	0.89 [0.60; 1.27]	0.793
Hemoglobin―mg/dL	12.9 [11.4; 14.0]	12.9 [11.3; 14.1]	0.898
Platelets―×10^3^/μL	188 [149; 228]	190 [149; 239]	0.713
Inflammation/infection markers―median [IQR]			
CRP―mg/dL	20.9 [6.00; 65.4]	45.2 [19.0; 93.6]	**0.004**
Ferritin―mg/dL	133 [66.0; 338]	284 [135; 598]	**0.005**
LDH―U/L	432 [393; 511]	542 [411; 851]	< **0.001**
Procalcitonin―ng/mL	0.12 [0.08; 0.32]	0.13 [0.07; 0.28]	0.522
Coagulation markers—median [IQR]			
D‐dimer―ng/mL	853 [700; 1656]	1200 [772; 1678]	0.419
Fibrinogen―mg/dL	659 (190)	692 (181)	0.272
Biochemistry and other markers―median [IQR]			
Glucose―mg/dL	118 [96.8; 167]	117 [101; 144]	0.757
Total bilirubin―mg/dL	0.60 [0.50; 1.00]	0.51 [0.32; 0.68]	**0.001**
Creatinin―mg/dL	0.96 [0.73; 1.29]	0.91 [0.76; 1.30]	0.956
eGFR―mL/min	59.5 [41.0; 78.5]	74.4 [44.6; 84.5]	0.168
Urea―mg/dL	50.5 [35.0; 65.8]	47.0 [33.0; 68.5]	0.947
SGPT―U/L	18.0 [13.0; 28.0]	35.5 [23.0; 50.2]	< **0.001**
SGOT―U/L	27.0 [18.0; 34.8]	20.6 [13.9; 38.9]	**0.042**
Alkaline phosphatase―U/L	85.0 [69.0; 105]	85.5 [62.8; 113]	0.500
Sodium―mmol/L	138 [135; 140]	137 [134; 139]	0.194
Potassium―mmol/L	4.10 [3.80; 4.43]	4.51 [4.08; 5.02]	< **0.001**
Ultrasensitive troponin―ng/mL	20.0 [14.0; 49.0]	19.0 [11.0; 29.5]	0.250

*Note:* Bold values indicate statistically significant *p* values (*p* < 0.05).

Abbreviations: CRP, C‐reactive protein; eGFR, estimated glomerular filtration rate; IQR, interquartile range; LDH, lactate dehydrogenase; SGOT, serum glutamic‐oxaloacetic transaminase; SGPT, serum glutamate pyruvate transaminase.

In the linear regression analysis (Table 4), differences in CRP, ferritin, and LDH levels remained significantly higher in the COVID‐19 group. Meanwhile, bilirubin and AST levels were slightly higher in the RSV group, whereas ALT and potassium levels were elevated in the COVID‐19 group. In the adjusted linear regression model, a difference favoring the COVID‐19 group was observed for CRP levels (24.55 mg/dL; 49.4 to −0.3, *p* = 0.053), LDH levels (164.68 U/L; 268.1–61.25, *p* = 0.002), and ALT levels (14.15 U/L; 24.75–3.55, *p* = 0.009).

**Table 4:** Linear regression analysis.

Characteristic	RSV *n* = 65	COVID‐19 *n* = 65	*p*
Death―No. (%)	5 (7.69)	13 (20)	0.075
Blood count―median [IQR]			
Absolute leukocytes―×10^3^/μL	7.40 [5.82; 10.8]	7.9 [5.4; 10.2]	0.980
Absolute neutrophils―×10^3^/μL	5.58 [3.53; 7.45]	6.01 [3.84; 8.3]	0.418
Inflammation/infection markers―median [IQR]			
CRP―mg/dL	18.8 [5.70; 63.4]	42.6 [19.2; 95.5]	**0.012**
Ferritin―mg/dL	115 [65.5; 306]	292 [198; 562]	**0.005**
LDH―U/L	438 [392; 511]	547 [406; 814]	**0.012**
Biochemistry and other markers―median [IQR]			
Total bilirubin―mg/dL	0.70 [0.50; 1.20]	0.53 [0.36; 0.74]	**0.015**
SGPT―U/L	16.0 [13.0; 28.0]	31.0 [23.0; 47.8]	< **0.001**
SGOT―U/L	27.0 [18.0; 35.0]	20.6 [13.7; 38.1]	0.073
Potassium―mmol/L	4.10 [3.80; 4.40]	4.47 [4.11; 4.93]	< **0.001**

*Note:* Bold values indicate statistically significant *p* values (*p* < 0.05).

Abbreviations: CRP, C‐reactive protein; IQR, interquartile range; LDH, lactate dehydrogenase; SGOT, serum glutamic‐oxaloacetic transaminase; SGPT, serum glutamate pyruvate transaminase.

## 4 Discussion

Our findings indicate that patients hospitalized with RSV tend to have more comorbidities, particularly COPD and CHF. This observation is consistent with another study conducted in Spain, in which 81% of RSV patients had cardiovascular conditions, 65% had endocrine‐metabolic disorders, and 46% had chronic lung diseases [8]. These comorbidities have been shown to significantly increase the risk of worse outcomes, including ICU admission and death [9, 10, 11].

Regarding clinical presentation, the length of hospitalization was similar between both groups, ranging from 5 to 10 days for RSV and from 4 to 12 days for COVID‐19. This is a factor that is often influenced by patients' baseline characteristics and, in general, tends to be around 10 days for both conditions [12, 13].

In our study, most patients with RSV had a normal chest X‐ray. However, previous studies have reported that, when a computed tomography (CT) scan is performed, the most common finding is a pattern of bilateral localized consolidation [14]. In contrast, our patients with COVID‐19 predominantly exhibited bilateral ground‐glass opacities, which is the most common radiographic presentation of pneumonia caused by this virus [15].

An unexpected finding was the difference in the use of antibacterial agents, with 75% of RSV patients receiving antibiotics compared to 41% of COVID‐19 patients. Although this latter figure is high, it is considerably lower than other studies, in one US‐based study, 94.1% of hospitalized older adults diagnosed with RSV received antibiotic therapy [10] despite the fact that the percentage of bacterial coinfection in patients with confirmed pneumonia ranges from 30% to 9% [16, 17]. In a similar context [18, 19], bacteraemia at admission has been reported in only 6.7% of cases [18], and in these instances, the source is usually not respiratory, suggesting that empirical antibiotic therapy may not be justified. In the case of COVID‐19 patients, between 30% [17] and 70% [19, 20] of hospitalized patients receive antibacterial treatment despite a coinfection rate is between 4% and 8% [17, 19, 20].

We believe that the lack of specific treatments for RSV may lead physicians to use antibiotics more frequently due to the perception of providing insufficient treatment. In the absence of specific and widely approved antiviral therapies for adult RSV infection, the mainstay of treatment remains supportive care. This includes oxygen supplementation, hydration, and physiologic support, with antiviral agents like ribavirin occasionally reserved for life‐threatening cases or for immunocompromised patients such as hematopoietic stem cell transplant recipients [21]. Guidelines from organizations such as the Infectious Diseases Society of America and the American Thoracic Society stress that the use of antibiotics should be reserved for situations where there is clear evidence or strong clinical suspicion of bacterial coinfection, to comply with antibiotic stewardship principles [21, 22]. Additionally, since RSV primarily affects older adults with comorbidities, physicians may suspect concomitant bacterial infections complicating the clinical presentation.

As is typical in viral infections, both diseases were associated with lymphopenia and normal total leukocyte counts, although slightly higher in RSV. COVID‐19 patients, on the other hand, showed higher levels of acute‐phase reactants (CRP, ferritin, and LDH) compared to RSV patients, which may be influenced by immune system senescence in RSV patients or the greater severity of SARS‐CoV‐2 infection.

Both infections can cause severe illness in at‐risk populations, which is why global prevention strategies, particularly vaccination, are critical. In our cohort, more than 80% of patients had received at least two doses of the COVID‐19 vaccine. These data align with the Spanish population, where universal vaccination recommendations and government funding have led to an 80% coverage rate [23]. In contrast, at the time of the study and prior to it, there were no official recommendations for RSV vaccination, and none of the patients had received an RSV vaccine. It is essential to optimize vaccination strategies for this population. Recently, the Spanish Ministry of Health conducted an evaluation of RSV vaccination in the adult population but, due to economic considerations, was unable to recommend its implementation [24]. In this cost‐utility analysis, vaccination coverage in patients aged 75 years or older was compared with no vaccination, yielding an incremental cost‐effectiveness ratio of €31,868.69 per QALY. The accepted threshold in this population within the Spanish context is €25,000 per QALY, placing the vaccination strategy slightly above the established limit. In light of our findings, it would be particularly relevant to perform this analysis focusing on adults over 70 years of age with comorbidities such as CHF or COPD. Moreover, it is appropriate to continue this line of research to better characterize the disease burden in different population subgroups and to inform future preventive strategies.

Importantly, this study contributes to the relatively scarce literature on RSV in hospitalized adults, particularly within the Spanish healthcare context.

Our study has some limitations. It is an observational, cross‐sectional study with inherent selection biases typical of this type of research. However, patients were consecutively selected during the study period without any prior selection criteria. The sample size was determined based on the exploratory nature of the study. The evaluation of chest X‐rays was performed by the attending physician without standardized diagnostic criteria for all patients, and a CT scan would have provided more accurate assessments of lung involvement. Additionally, we lack data on how many patients who received antibiotics ultimately had confirmed bacterial coinfections. Nevertheless, this is a pioneering study—we have not identified other series comparing these conditions in our setting, and our findings are important for improving the management and prevention of RSV disease in adults.

**Figure 1:**
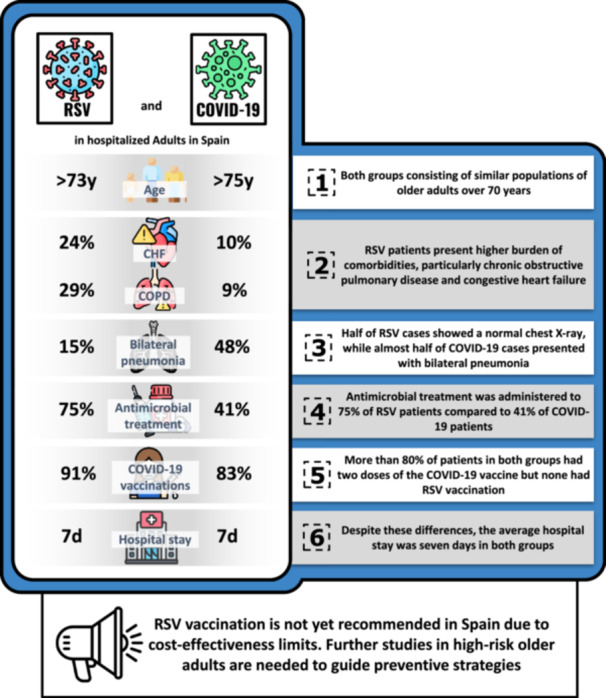
RSV and COVID‐19: a side‐by‐side look at hospitalized adults in Spain.

In conclusion, there are significant differences between patients hospitalized with RSV and those with COVID‐19. RSV patients, who are predominantly older adults with multiple comorbidities, showed a higher prevalence of CHF and COPD, which may contribute to a greater use of antibiotics. Although hospitalization duration and oxygen therapy requirements were similar between both groups, radiographic patterns and inflammatory markers differed significantly, with COVID‐19 patients presenting higher systemic inflammation and greater lung involvement. The high prescription rate of antibiotics in both groups underscores the need to review treatment strategies and antimicrobial stewardship in viral infections. Lastly, further research is essential to evaluate specific treatments for RSV and optimize preventive interventions in adults over 70 years of age with CHF and COPD (Figure 1). However, due to the retrospective design, limited sample size, and potential for residual confounding, these associations should be interpreted with caution. Moreover, the multiplicity of analyses may increase the risk of type I error, and the lack of longitudinal follow‐up limits understanding of postdischarge outcomes. Further prospective, multicentre studies with larger cohorts are needed to validate these findings and inform evidence‐based management and prevention policies for RSV in adults.

## Author Contributions

**Jose‐Reynaldo Homen Fernandez:** writing – original draft (lead), writing – review and editing (equal), project administration (lead), investigation (equal), methodology (supporting), visualization (lead). **Inés Armenteros:** writing – review and editing (equal), investigation (equal), conceptualization (supporting). **Adrián Valls Carbó:** data curation (lead), writing – original draft (supporting), review and editing (equal), methodology (lead), formal analysis (lead). **Julia Barrado:** writing – review and editing (equal). **Carolina Olmos‐Mata:** writing – review and editing (equal). **Ana Muñoz:** writing – review and editing (equal). **Juncal Pérez‐Somarriba:** writing – review and editing (equal). **Noemí Cabello:** writing – review and editing (equal), conceptualization (supporting). **María José Núñez:** writing – review and editing (equal). **Vicente Estrada:** conceptualization (lead), writing – review and editing (equal), supervision (lead), validation (lead). All authors approved the final manuscript.

## Ethics Statement

This work has been reviewed and accepted by the Ethics Committee for Research Ethics with Medicines of the Hospital Clínico San Carlos who approve that the requirements established in the current legislation—Royal Decree 1090/2015—were met. It complies with the GCP rules (CPMP/ICH/135/95) and with the current legislation that regulates its operation, and that the composition of the Hospital Clínico San Carlos Committee taking into account that in the event that any member participates in the project or declares any conflict of interest will not have participated in the evaluation or in the opinion of the application for authorization of the project.

## Conflicts of Interest

The authors declare no conflicts of interest.

## References

[1] Global Burden of Disease Study 2019 (GBD 2019) , *Global Burden of Disease Collaborative Network* (GBD, 2020).

[2] S. Safiri , A. Mahmoodpoor , A. A. Kolahi , et al., “Global Burden of Lower Respiratory Infections During the Last Three Decades,” Frontiers in Public Health 10 (2023): 1028525. 36699876 10.3389/fpubh.2022.1028525 PMC9869262

[3] C. Troeger , B. Blacker , I. A. Khalil , et al., “Estimates of the Global, Regional, and National Morbidity, Mortality, and Aetiologies of Lower Respiratory Infections in 195 Countries, 1990–2016: A Systematic Analysis for the Global Burden of Disease Study 2016,” Lancet Infectious Diseases 18 (2018): 1191–1210. 30243584 10.1016/S1473-3099(18)30310-4 PMC6202443

[4] J. S. Nguyen‐Van‐Tam , M. O'Leary , E. T. Martin , et al., “Burden of Respiratory Syncytial Virus Infection in Older and High‐Risk Adults: A Systematic Review and Meta‐Analysis of the Evidence From Developed Countries,” European Respiratory Review 31 (2022): 220105. 36384703 10.1183/16000617.0105-2022 PMC9724807

[5] A. R. Branche , L. Saiman , E. E. Walsh , et al., “Incidence of Respiratory Syncytial Virus Infection Among Hospitalized Adults, 2017–2020,” Clinical Infectious Diseases 74 (2022): 1004–1011. 34244735 10.1093/cid/ciab595

[6] S. J. Olsen , A. K. Winn , A. P. Budd , et al., “Changes in Influenza and Other Respiratory Virus Activity During the COVID‐19 Pandemic – United States, 2020–2021,” Morbidity and Mortality Weekly Report 70 (2021): 1013–1019. 34292924 10.15585/mmwr.mm7029a1 PMC8297694

[7] N. Zhang , L. Wang , X. Deng , et al., “Recent Advances in the Detection of Respiratory Virus Infection in Humans,” Journal of Medical Virology 92 (2020): 408–417. 31944312 10.1002/jmv.25674 PMC7166954

[8] M. Haeberer , M. Mengel , R. Fan , et al., “RSV Risk Profile in Hospitalized Adults and Comparison With Influenza and COVID‐19 Controls in Valladolid, Spain, 2010–2022,” Infectious Diseases and Therapy 13 (2024): 1983–1999. 39033476 10.1007/s40121-024-01021-1 PMC11343947

[9] L. Kim , B. Cikesh , P. D. Kirley , et al., “746. Characteristics of Respiratory Syncytial Virus (RSV) Infection Among Hospitalized Adults, United States, 2014–2017,” Open Forum Infectious Diseases 5 (2018): S268.

[10] B. Ackerson , H. F. Tseng , L. S. Sy , et al., “Severe Morbidity and Mortality Associated With Respiratory Syncytial Virus Versus Influenza Infection in Hospitalized Older Adults,” Clinical Infectious Diseases 69 (2019): 197–203. 30452608 10.1093/cid/ciy991 PMC6603263

[11] F. P. Havers , M. Whitaker , M. Melgar , et al., “Characteristics and Outcomes Among Adults Aged ≥ 60 Years Hospitalized With Laboratory‐Confirmed Respiratory Syncytial Virus – RSV‐NET, 12 States, July 2022–June 2023,” Morbidity and Mortality Weekly Report 72 (2023): 1075–1082. 37796742 10.15585/mmwr.mm7240a1 PMC10564327

[12] A. Colosia , J. Costello , K. McQuarrie , K. Kato , and K. Bertzos , “Systematic Literature Review of the Signs and Symptoms of Respiratory Syncytial Virus,” Influenza and Other Respiratory Viruses 17 (2023): e13100. 36824394 10.1111/irv.13100 PMC9899685

[13] Y. Alimohamadi , E. M. Yekta , M. Sepandi , M. Sharafoddin , M. Arshadi , and E. Hesari , “Hospital Length of Stay for COVID‐19 Patients: A Systematic Review and Meta‐Analysis,” Multidisciplinary Respiratory Medicine 17, no. 1 (2022): 856, 10.4081/mrm.2022.856. 36117876 PMC9472334

[14] M. Riccò , S. Corrado , S. Palmieri , and F. Marchesi , “Respiratory Syncytial Virus: A Systematic Review and Meta‐Analysis of Tomographic Findings (2000–2022),” Children 10 (2023): 1169. 37508666 10.3390/children10071169 PMC10378054

[15] V. Ojha , A. Mani , N. N. Pandey , S. Sharma , and S. Kumar , “CT in Coronavirus Disease 2019 (COVID‐19): A Systematic Review of Chest CT Findings in 4410 Adult Patients,” European Radiology 30 (2020): 6129–6138. 32474632 10.1007/s00330-020-06975-7 PMC7261039

[16] M. Jeannoël , G. Lina , J. P. Rasigade , B. Lina , F. Morfin , and J. S. Casalegno , “Microorganisms Associated With Respiratory Syncytial Virus Pneumonia in the Adult Population,” European Journal of Clinical Microbiology & Infectious Diseases 38 (2019): 157–160. 30353485 10.1007/s10096-018-3407-3 PMC7101617

[17] P. Hedberg , N. Johansson , A. Ternhag , L. Abdel‐Halim , J. Hedlund , and P. Nauclér , “Bacterial Co‐Infections in Community‐Acquired Pneumonia Caused by SARS‐CoV‐2, Influenza Virus and Respiratory Syncytial Virus,” BMC Infectious Diseases 22 (2022): 108. 35100984 10.1186/s12879-022-07089-9 PMC8802536

[18] E. Sano , B. Chang , W. Sieling , et al., “Bacteremia in Adults Admitted From the Emergency Department With Laboratory‐Confirmed Respiratory Syncytial Virus,” Journal of Emergency Medicine 62 (2022): 216–223. 35031172 10.1016/j.jemermed.2021.10.019

[19] T. M. Rawson , L. S. P. Moore , N. Zhu , et al., “Bacterial and Fungal Co‐Infection in Individuals With Coronavirus: A Rapid Review to Support COVID‐19 Antimicrobial Prescribing,” Clinical Infectious Diseases 71 (2020): 2459–2468. 32358954 10.1093/cid/ciaa530 PMC7197596

[20] B. J. Langford , M. So , S. Raybardhan , et al., “Antibiotic Prescribing in Patients With COVID‐19: Rapid Review and Meta‐Analysis,” Clinical Microbiology and Infection 27 (2021): 520–531. 33418017 10.1016/j.cmi.2020.12.018 PMC7785281

[21] F. Alfano , T. Bigoni , F. P. Caggiano , and A. Papi , “Respiratory Syncytial Virus Infection in Older Adults: An Update,” Drugs & Aging 41 (2024): 487–505. 38713299 10.1007/s40266-024-01118-9 PMC11193699

[22] M. Woodhead , F. Blasi , S. Ewig , et al., “Guidelines for the Management of Adult Lower Respiratory Tract Infections ‐ Full Version,” Clinical Microbiology and Infection 17 (2011): E1–E59. 10.1111/j.1469-0691.2011.03672.x PMC7128977 21951385

[23] Ministerio de Sanidad , “Cuadro de mando resumen de datos de vacunación [Summary Dashboard of Vaccination Data],” accessed July 1, 2024, https://www.sanidad.gob.es/areas/alertasEmergenciasSanitarias/alertasActuales/nCov/pbiVacunacion.htm.

[24] Grupo de Trabajo vacunación frente VRS en población adulta de la Ponencia de Programa y Registro, de Vacunaciones, “Evaluación de La Vacunación Frente a VRS En La Población Adulta [Evaluation of RSV Vaccination in the Adult Population. Public Health Commission of the Interterritorial Council of the National Health System],” (Ministry of Health, 2024).

